# Patient values in patient-provider communication about participation in early phase clinical cancer trials: a qualitative analysis before and after implementation of an online value clarification tool intervention

**DOI:** 10.1186/s12911-024-02434-1

**Published:** 2024-02-02

**Authors:** Liza G. G. van Lent, Mirte van der Ham, Maja J. A. de Jonge, Eelke H. Gort, Marjolein van Mil, Jeroen Hasselaar, Carin C. D. van der Rijt, Jelle van Gurp, Julia C. M. van Weert

**Affiliations:** 1 Department of Medical Oncology, Erasmus MC Cancer Institute, Erasmus University Medical Centre, Rotterdam, the Netherlands; 2 Department of Medical Oncology, UMC Utrecht Cancer Centre, Utrecht, the Netherlands; 3 Department of Medical Oncology and Clinical Pharmacology, Antoni Van Leeuwenhoek, the Netherlands Cancer Institute, Amsterdam, the Netherlands; 4 Department of Pain, Anaesthesiology and Palliative Care, Radboud University Medical Centre, Nijmegen, the Netherlands; 5 Department of IQ Healthcare, Radboud University Medical Centre, Nijmegen, the Netherlands; 6 Department of Communication Science, Amsterdam School of Communication Research (ASCoR) and University of Amsterdam, Amsterdam, the Netherlands

**Keywords:** Cancer, Decision Support Techniques, Health Communication, Shared Decision Making, Value Clarification, Intervention, Digital Tool, Communication Skills Training

## Abstract

**Background:**

Patients with advanced cancer who no longer have standard treatment options available may decide to participate in early phase clinical trials (i.e. experimental treatments with uncertain outcomes). Shared decision-making (SDM) models help to understand considerations that influence patients’ decision. Discussion of patient values is essential to SDM, but such communication is often limited in this context and may require new interventions. The OnVaCT intervention, consisting of a preparatory online value clarification tool (OnVaCT) for patients and communication training for oncologists, was previously developed to support SDM. This study aimed to qualitatively explore associations between patient values that are discussed between patients and oncologists during consultations about potential participation in early phase clinical trials before and after implementation of the OnVaCT intervention.

**Methods:**

This study is part of a prospective multicentre nonrandomized controlled clinical trial and had a between-subjects design: pre-intervention patients received usual care, while post-intervention patients additionally received the OnVaCT. Oncologists participated in the communication training between study phases. Patients’ initial consultation on potential early phase clinical trial participation was recorded and transcribed verbatim. Applying a directed approach, two independent coders analysed the transcripts using an initial codebook based on previous studies. Steps of continuous evaluation and revision were repeated until data saturation was reached.

**Results:**

Data saturation was reached after 32 patient-oncologist consultations (i.e. 17 pre-intervention and 15 post-intervention). The analysis revealed the values: hope, perseverance, quality or quantity of life, risk tolerance, trust in the healthcare system/professionals, autonomy, social adherence, altruism, corporeality, acceptance of one’s fate, and humanity. Patients in the pre-intervention phase tended to express values briefly and spontaneously. Oncologists acknowledged the importance of patients’ values, but generally only gave ‘contrasting’ examples of why some accept and others refuse to participate in trials. In the post-intervention phase, many oncologists referred to the OnVaCT and/or asked follow-up questions, while patients used longer phrases that combined multiple values, sometimes clearly indicating their weighing.

**Conclusions:**

While all values were recognized in both study phases, our results have highlighted the different communication patterns around patient values in SDM for potential early phase clinical trial participation before and after implementation of the OnVaCT intervention. This study therefore provides a first (qualitative) indication that the OnVaCT intervention may support patients and oncologists in discussing their values.

**Trial registration:**

Netherlands Trial Registry: NL7335, registered on July 17, 2018.

**Supplementary Information:**

The online version contains supplementary material available at 10.1186/s12911-024-02434-1.

## Background

Decision support technologies, or decision aids are evidence-based tools designed to support patients in reaching informed, value-based choices [1, 2] and have been widely recommend to assist patients in making difficult decisions. Patient values are desires, goals or beliefs that patients find important [3, 4] and are related to the perspectives that these patients have on the ‘good life’ [5]. The Ottawa Decision Support framework [6, 7] considers clarifying patient values a key part of decision support (technologies). Recent systematic reviews confirmed that “unclear values” are an important decisional limitation for patients [8]. Decision aids can improve decisional quality [9] as well as patient‐clinician communication and congruence between patients’ informed values and their chosen options [10]. Specifically, exposure to a decision aid with explicit value clarification resulted in a higher proportion of patients choosing an option congruent with their values [11]. Naturally, the International Patient Decision Aids Standards (IPDAS) strongly recommends the inclusion of value clarification in decision aids [1].

If value clarification methods (e.g. in decision aids) are used before and during a consultation, this could help patients to become more aware of their values and facilitate a shared decision-making (SDM) process [12]. SDM is “an approach where clinicians and patients share the best available evidence when faced with the task of making decisions, and where patients are supported to consider options, to achieve informed preferences” [13], and is highly recommended for decision-making between patients and providers [14, 15], especially when a choice is value-sensitive. Widely used SDM models consider eliciting patient values and preferences a key part of health- and treatment-related decision-making [12, 16, 17]. Having a discussion (e.g. during a doctor-patient consultation) on patient values is particularly important because patients rarely reach clarity of values and preferences without the help of others [18]. Moreover, discussing patient values can enhance patients’ involvement in the SDM process [19]. This aligns well with patient-centred care which means “providing care that is respectful of and responsive to individual patient preferences, needs, and values and ensuring that patient values guide all clinical decisions” [20, 21]. Indeed, one of SDM’s main goals is to deliver care in line with patients’ personal values [22, 23]. Discussing patient values becomes even more important when there is no clear ‘best’ or ‘worst’ option in the upcoming decision.

A decision that is – ideally – pre-eminently based on patient values, is the decision whether or not to participate in oncological early phase clinical trials, i.e. trials on experimental treatments with unknown outcomes in terms of e.g. effectiveness, toxicity and survival. Some patients who decide to participate in early phase clinical trials hope for clinical benefit [24] even while realizing that this chance may be small [25]. Patients could also decide not to participate but rather to focus on alleviating their existing symptoms and complaints by means of palliative care. Patients who participate in early phase clinical trials however may also use palliative care services in addition to the experimental trial with its many uncertainties. This complex context creates a difficult decision with no really ‘best’ option and consequently has major impact on the patients’ life. Integrating patient values into the SDM process could help ensure that patients spend the limited time towards the end of their life in the way that they want. In addition to hope, a previous systematic review [24] and a qualitative interview study [26] of our team found that important patient values impacting the decision upon early phase clinical trial participation are, amongst others, quality and quantity of life, acceptance (e.g. of their prospective death) and body preservation (e.g. maintaining fitness of the body, or preventing destruction of bodily senses).

However, while previous systematic reviews support the idea that SDM for early phase clinical trials needs to address both medical-technical information and patients’ values and preferences [27, 28], discussions about participation in early phase clinical trials tend to focus on explaining complex medical-technical information to patients [27, 28]. In this sense, oncologists currently appear to use an ‘informative model’ [29]. As these studies illustrate the currently limited integration of patient values, it remains important to support such a discussion. Aligning information with the subjective preferences of patients with advanced cancer may prevent unmet information needs [30]. However, moving towards an ‘interpretative model’ [29], in which oncologists take on the role of counsellor to help patients articulate and weigh their values, has its own challenges. Even when patients are empowered to overcome the difficulties of sharing their values [27], the challenge for oncologists remains to work with the patient during the consultation to appropriately clarify these values and how they relate to each other. Based on the literature on SDM and decision aids, a new intervention centred on value clarification could potentially provide a solution for these problems.

Following the Medical Research Council (MRC) framework for complex interventions [31, 32], we systematically developed the theory- and evidence-based OnVaCT intervention [33], consisting of an online value clarification tool (OnVaCT) for patients and communication training for oncologists. Together, these aimed to support patients and oncologists in clarifying and discussing patients’ personal values. In practice, we hypothesized that the OnVaCT intervention could lead to changes in the communication patterns, for instance by means of a (more) clear, explicit and/or extensive discussion on patient values and their relative weighing for individual patients. With this study, we want to unravel how the implementation of the OnVaCT intervention is actually reflected in the discussion of patient values in clinical practice, i.e. in doctor-patient consultations focusing on SDM for potential early phase clinical trials. More specifically, this study qualitatively analysed (in a between-subjects design, i.e. pre-intervention and post-intervention phase) the following research questions:

1. Which patient values are discussed (in context with each other) between patients and oncologists during consultations about potential early phase clinical trial participation before and after the implementation of the OnVaCT intervention?
2. How are patient values discussed between patients and oncologists during consultations about potential early phase clinical trial participation before and after the implementation of the OnVaCT intervention?

## Method

### Design

This study is a qualitative (content) analysis that is part of a larger prospective multicentre nonrandomized controlled clinical trial on SDM for early phase clinical trial participation that makes use of mixed-methods (Netherlands Trial Registry: NL7335) [34]. This study had a between-subjects design: patients included in the pre-intervention phase (i.e. before implementation of the OnVaCT intervention) functioned as ‘control group’ within current standard practice/usual care, while patients in the post-intervention phase (i.e. after implementation of the OnVaCT intervention) functioned as the ‘intervention group’ and were asked to use the OnVaCT before their consultation. Oncologists from three hospitals with large early phase clinical trial units in the Netherlands (i.e. Erasmus MC, Rotterdam; Netherlands Cancer Institute–Antoni van Leeuwenhoek, Amsterdam; and UMC Utrecht) participated in the communication training between the two study phases. The final OnVaCT intervention is summarized in Table 1 and described in detail elsewhere [33, 35]. Standards for Reporting Qualitative Research [36] were used to report the qualitative analysis.

**Table 1:** Summary of the OnVaCT intervention [33, 35]

The OnVaCT intervention was systematically developed following the Medical Research Council (MRC) framework for complex interventions, in co-creation with the end-users (i.e. patients and oncologists).
**The preparatory OnVaCT** for patients was developed by a professional company, specialized in designing and developing interactive, playful, digital ‘learning’ tools such as serious games and interactive experience tools. The OnVaCT was built upon five characters or avatars – all hypothetical patients facing the decision whether to participate in early phase clinical trials – who each consisted of three short narratives in which different (combinations of) patient values were addressed. The narratives and values were derived from a previous systematic review and interview study, and aligned well with the initial codebook for this study (Table 1). In the OnVaCT, patients were supported to actively reflect on their personal values in light of the upcoming consultation by means of questions (e.g. “how do you feel about that?”).
**The communication training** for oncologists was developed with and led by an experienced psychologist who is an expert in providing communication skills training to medical students and medical specialists. The training consisted of three parts: (1) a web lecture (approx. 15 minutes) regarding SDM during consultations about potential participation in early phase clinical trials; (2) an individual feedback session during which oncologists received personalized feedback on one of their pre**-**intervention recordings, which they were asked to watch and assess using the SDM steps beforehand (approx. 30 minutes and 1 hour preparation); and (3) a concluding group training, that focused on making the (results from using the) OnVaCT and patient values discussable with the patients by using SDM steps (approx. 2 hours).

### Participants

The inclusion criteria were: diagnosed with advanced cancer and eligible for first participation in an early phase clinical trial (i.e. phase I or phase I/II); aged 18 years or older; sufficient command of the Dutch language; and written informed consent. Exclusion criteria were: cognitive impairment according to the medical record; no access to the Internet to fill out the online questionnaires (and use the OnVaCT); or participation in another part of the project (*N* = 13) [26].

### Procedure

From February 18, 2019 up to December 18, 2020, patients were approached to participate in the pre-intervention phase (i.e. before implementation of the OnVaCT intervention). The study was put on-hold between March 16 and May 20, 2020 due to restrictive measures regarding the COVID-19 pandemic. Patients were approached to participate in the post-intervention phase between January 26, 2021 and August 31, 2022. Eligible patients were called by a nurse practitioner, trial secretary or researcher from their own hospital. Patients who verbally agreed, were sent an e-mail with the patient information and consent form by a researcher/assistant, and were called again to ask for preliminary consent. Written informed consents were signed and collected directly before the consultation. The consultation was (video- or) audiotaped. Local trial monitors later collected additional data regarding patients’ decision and their medical status from electronic patient records.

The OnVaCT intervention was implemented between December 18, 2020 and February 1, 2021. During this period, participating oncologists followed the communication training and the procedures (e.g. patient package) were adjusted. In the post-intervention phase, patients were sent the link to the OnVaCT via e-mail before their initial consultation, and were asked to use it in preparation to the consultation. Whether patients had used the OnVaCT prior to the consultation was verified in the transcripts and by checking whether they had filled out a questionnaire about the OnVaCT (results of this questionnaire will be reported elsewhere).

### Sample

Starting with the pre-intervention data, we selected one recording for each participating oncologist who had at least one available recording in both study phases during every round of coding. We primarily aimed for an equal division of patients who accepted and refused trial participation (in total and per oncologist), and secondarily for a diverse group in terms of gender, age, and primary tumour location. We analysed pre-intervention consultations until saturation of themes was reached. Then, the entire procedure was repeated for the post-intervention phase. To avoid drop-out bias (i.e. to not miss relevant views and values from patients who could or would not comply to use the intervention), we applied an intention-to-treat approach [37]: patients included in the post-intervention phase were analysed as such, regardless of whether they had actually used the OnVaCT.

### Data analysis

The qualitative software NVivo for Windows (release 1.4) was used. We applied a directed approach (which is both inductive and deductive) to thematically analyse the recordings [38]. An initial coding scheme with (sub)themes (see Table 2) was deductively created based on the findings from our previous systematic review [24] and interview study [26]. During the data analysis, the coding scheme was inductively adjusted (e.g. additions and/or omissions were made) based on the actual content of the communication (see below).

**Table 2:** Initial codebook

**Patient values and subthemes**	**Definition** (based on review and interview study)
**Hope** • Hope for personal benefit • Having an optimistic attitude	The desire, belief or feeling that participating in an early phase clinical cancer trial will lead to personal benefit (such as tumour shrinkage or prolongation of life). Hope and optimism are often used as synonyms
**Perseverance** • Wanting to ‘fight/battle’ cancer • Wanting to have tried everything	The desire to keep fighting/battling (or to keep living), despite having little treatment options or a bad prognosis
**Quality or quantity of life** • Quality of life • Quantity of life	The preferences someone has regarding the balance and potential trade-off between living as long as possible and maintaining quality of life
**Risk tolerance** • Wanting to take a gamble/risk (by participating in a trial) • Regular/close follow-up	The willingness or ability someone has to accept risks or take gambles
**Trust in the healthcare system or healthcare professional** • Trust in medicine • Communicating trust in the healthcare professional	The belief that a healthcare professional or institution (including his/her judgement and endorsement) is good, sincere, and/or honest and that he/she would not willingly trick or harm someone
**Autonomy** • Gaining a sense of control by participating • Wanting to make a decision for oneself • Wanting to be/stay/act independent	The desire or ability to act and make decisions without being controlled by and/or dependent on others in the context of a life-limiting disease
**Social adherence** • Feeling pressure from others (family/friends) • Wanting to follow the wishes of others (family/friends)	The desire or willingness to behave according to the expectations, values or attitudes of others (especially family) regarding participation in an early phase clinical cancer trial
**Altruism** • Wanting to help future patients • Wanting to help research/medicine	The desire or willingness to accept or decline participation in an early phase clinical cancer trial motivated by the care for others (including wanting to help research/medicine), even though it does not necessarily lead to personal benefit
**Body preservation** • Maintaining a healthy lifestyle • Relaxing/experiencing pleasure (unhealthy behaviour does not matter anymore) • (Dis)trusting one’s body	The relation with the body changes when being diagnosed with cancer. What people find most important; creating the optimal environment to succeed treatment by living healthy or seeing the body as a medium for enjoyment has a determining role in the decision-making process
**Accepting one’s fate** • Religious faith and/or being guided by God • Spiritual faith	The willingness or ability someone has to accept the things, especially bad things such as nearing death and/or worsening complaints, that will happen to him/her. In this sense, religion and spirituality may serve as means to help accept such a fate
**Humanity** • Not wanting to be a ‘guinea pig’ • Wanting to be treated as human, rather than a patient • Wanting others to show interest in them (and their illness/family/etc.)	The desire to be, feel, or be treated as a person rather than a god, an animal, a machine, or a patient
**Attempt of the doctor to start discussion/deliberation of value(s)**	An effort the oncologist makes to start or continue a discussion or deliberation with the patient regarding his/her values (e.g. with questions or examples). N.B. This is not considered a ‘patient value’ but was included in the codebook to better enable the unravelling of how these values were discussed

Selected recordings were transcribed verbatim and analysed by two coders with a background in communication (LL) and health sciences (MH). The data analysis of the transcripts was an ongoing, iterative process, with each transcript being double-coded by the two researchers independently. After every transcript, they compared their findings and resolved discrepancies in the codes by means of discussion. The progress of the coding process (including discrepancies and findings) was also discussed during regular meetings with two other researchers with a health communication (JW) and medical ethics (JG) background. If needed, adjustments to the codebook were made after establishing mutual agreement between the four researchers. Although not quantitatively verified, the two independent coders reached a good level of agreement after the first five transcripts, with generally no major discrepancies between them for the remaining consultations. Findings were peer-reviewed by two researchers/oncologists with a palliative care (CR) and early phase clinical trial (MJ) background. These steps of continuous evaluation and revision were repeated until data saturation was reached (i.e. no new themes arose) for both study phases separately. Supplementary Table 1 shows the final codebook.

When data saturation was reached for both study phases, a more ‘inductive’ phase of the analysis started in which the coders took a closer look at the encoded phrases per value to assess which values and subthemes were (not) discussed, the possible links between values, and which subthemes could be recognized into how these values were discussed by patients and oncologists. To enable analysis of values discussed in context with each other, each value was encoded together with the entire phrase in which it occurred, not to lose relevant sentences/questions/replies that preceded or followed the mentioned value. The two coders compared and discussed their labels and the observed links between different values, also together with the study team, until consensus was reached. Finally, the pre-intervention and post-intervention findings (that were in two separate NVivo files) were compared to each other by first comparing the patient values *that* were discussed (in context with each other) in the pre-intervention phase to those discussed (in context with each other) in the post-intervention phase, and then *how* these values were discussed in these two phases.

## Results

Table 3 shows the characteristics of the selected 32 patients (i.e. seventeen pre-intervention and fifteen post-intervention) divided across nine oncologists (range: 2–4 recordings per oncologist). Patients were mostly male (*N* = 19) and between 36 and 78 years old. The consultations lasted between 28 and 53 min. In both study phases, approximately half of the patients decided to participate in an early phase clinical trial. Two post-intervention patients did not use the OnVaCT prior to the consultation. In the following paragraphs, we will first describe which of the patient values were discussed and then elaborate on the connections that were made between the values (RQ1), and then how oncologists and patients discussed patient values (RQ2). In each paragraph, the results from the pre-intervention and post-intervention phase are described consecutively to explore how the implementation of the OnVaCT intervention was reflected in these discussions.

**Table 3:** Patient characteristics

Subject	Decided to participate (yes/no)	Gender	Age	Primary tumour location	Oncologist	Duration of consultation (minutes)	Used OnVaCT before consultation (yes/no)
**Pre-intervention, usual care/before implementation of the OnVaCT**							
**01**	No	Female	59	Colorectal/anal	**A**	28	N/A
**02**	No	Male	66	Oesophageal/stomach	**B**	49	N/A
**03**	No	Male	69	Oesophageal/stomach	**C**	41	N/A
**04**	No	Female	62	Urinary tract	**D**	33	N/A
**05**	Yes	Female	75	Gynaecological	**C**	45	N/A
**06**	Yes	Male	66	Urinary tract	**D**	53	N/A
**07**	Yes	Male	58	Lung/mesothelioma	**B**	20	N/A
**08**	Yes	Male	72	Urinary tract	**A**	38	N/A
**09**	No	Female	50	Gynaecological	**E**	35	N/A
**10**	Yes	Male	54	Melanoma/skin	**F**	51	N/A
**11**	Yes	Female	39	Other	**G**	39	N/A
**12**	No	Male	53	Colorectal/anal	**H**	32	N/A
**13**	Yes	Male	61	Oesophageal/stomach	**E**	32	N/A
**14**	No	Female	53	Colorectal/anal	**F**	50	N/A
**15**	Yes	Male	78	Lung/mesothelioma	**H**	36	N/A
**16**	Yes	Male	72	Lung/mesothelioma	**I**	34	N/A
**17**	No	Male	72	Colorectal/anal	**I**	28	N/A
**Post-intervention, after implementation of the OnVaCT**							
**18**	No	Male	58	Other	**C**	42	Yes
**19**	No	Female	69	Colorectal/anal	**B**	43	Yes
**20**	Yes	Female	62	Colorectal/anal	**B**	32	No
**21**	No	Male	76	Other	**A**	29	Yes
**22**	Yes	Male	51	Other	**A**	29	Yes
**23**	Yes	Male	62	Oesophageal/stomach	**D**	44	Yes^a^
**24**	Yes	Female	42	Gynaecological	**C**	45	Yes
**25**	No	Male	73	Urinary tract	**D**	45	Yes^a^
**26**	Yes	Female	60	Oesophageal/stomach	**E**	30	Yes^a^
**27**	No	Female	50	Colorectal/anal	**H**	30	Yes^a^
**28**	No	Male	74	Oesophageal/stomach	**F**	28	Yes^a^
**29**	No	Male	68	Urinary tract	**G**	41	Yes^a^
**30**	Yes	Female	36	Gynaecological	**F**	28	No^b^
**31**	No	Male	65	Colorectal/anal	**I**	34	Yes^a^
**32**	Yes	Female	55	Colorectal/anal	**I**	50	Yes

^a^ These patients filled out questions about the OnVaCT for another part of the project, but whether they had actually used it was not explicitly mentioned during the consultation

^b^ This patient did not fill out questions about the OnVaCT for another part of the project, and whether she had actually used it was not explicitly mentioned during the consultation

### Which patient values were discussed

The consultations revealed the values hope, perseverance, quality or quantity of life, risk tolerance, trust, autonomy, social adherence, altruism, corporeality, accepting one’s fate, and humanity. Table 4 provides a more extensive description with quotes for each of the values that were used during consultations.

**Table 4:** Description of each patient value that was discussed in the pre-intervention and post-intervention phase

Patient values	Summary from current study
Hope	In both study phases, patients regularly expressed to hope for personal benefit by participating in an early phase clinical trial. Some patients thereby called an early phase clinical trial their “last straw” (subject 10, pre-intervention) or “nothing ventured, nothing gained” (subject 03, pre-intervention), whereas others directly mentioned the hope to personally benefit. Often, these expressions were paired with a concession or nuance: they hope to benefit from trial participation, but if not, then at least they will have tried. This thereby seemed to relate to perseverance, which will be described in more detail below. Hope for personal benefit also seemed to relate to quality or quantity of life: patients hope the treatment prolongs their life and/or makes the physical condition better. Some patients stated that they hope for a possibility for experimental treatment, or that they want to keep hope, but they do not always mention what exactly they are hoping for. Patients did not explicitly say that they have an optimistic attitude, but in some cases their expressions seemed to indicate that they have a positive outlook on life, for instance: “as long as […] the sun shines, you have to make the most of every day” (subject 19, post-intervention). One patient had hoped for more (i.e. better results from early phase clinical trials) than what was offered during the consultation
Perseverance	With regards to perseverance, patients did not (literally) mention that they want to have tried ‘everything’ or to keep fighting. Occasionally, patients mentioned that they do not want to give up or that they are “not a quitter” (subject 04, pre-intervention). Instead, in both study phases, several patients appeared to consider it important to ‘stay busy’ by participating in an early phase clinical trial instead of ‘doing nothing’. This sometimes seemed to relate to their hope for benefit from an early phase clinical trial (‘nothing ventured is nothing gained’). Generally, it seemed that these patients do not want to have the feeling that they could have done something, but did not try it
Quality or quantity of life	The chance that participation in an early phase clinical trial offers a chance or prolongation of life, was a relevant consideration for many patients in both the pre-intervention and the post-intervention phase. Patients sometimes elaborated on this by stating their goals in life (e.g. wanting to become a grandparent). Other patients stated that they wanted to live longer, but not at the expense of their quality of life or “if [they] would become more ill” (subject 02, pre-intervention), because that would be “a waste of [the extra] time” (subject 18, post-intervention). Some patients explicitly asked about their life expectancy, others stated that they do not want to know how long they have left to live. Generally, if patients mentioned ‘quality of life’ they referred to their physical condition and complaints, but it often remained unclear what a ‘high-quality’ life would look like to them. Some patients mentioned what they “try to do the things [they] want to do” (subject 06, pre-intervention) in terms of e.g. hobbies, tasks and events: “I’ve always been able to work, I like that. I want to continue doing so” (subject 10, pre-intervention). Some patients currently experienced a good quality of life, whereas this applied less to others (e.g. due to side effects from previous treatment). A few patients said they experienced psychological issues or had sought psychological support
Risk tolerance	Many patients acknowledged the uncertainty of (potential benefits from) participation in early phase clinical trials, because “you never know beforehand what you’re getting yourself into” (subject 04, pre-intervention). Some patients explicitly stated that they are willing to accept the risks of experimental treatments, while others mentioned that they are scared (e.g. of potential side-effects or ‘scary procedures’ such as biopsies) or that they are still debating whether the risk is worth it for them. It appeared as though other values can help patients in tolerating risks. For instance, “On the one hand, you can be excited to start trying that [trial participation]. If you don’t try it, you’ll have nothing anyway. But on the other hand, I also think, what will be done to you?” (relative of subject 02, pre-intervention). In that sense, the patients who were willing to tolerate risks and uncertainty seemed to be the ones who strongly value hope, perseverance and/or quantity of life. Furthermore, some patients mentioned that they trust the healthcare system or professional to adequately deal with these risks. Patients occasionally indicated their concerns for potential side-effects by repeatedly asking questions about those. The desire for regular or close follow-up in relation to participating in an early-phase clinical trial was rarely mentioned by patients
Trust in the healthcare system or healthcare professional	In both the pre-intervention and the post-intervention, some patients spontaneously acknowledged their belief that the healthcare professional or system will not willingly harm them, or will help them if something goes wrong (e.g. if they experience side effects from the early phase clinical trial). As explained above, trust thereby seemed to help patients in tolerating risks. For instance, a patient used trust as a means to control her fear for biopsies: “So yes, then I will take the fact that I think such a biopsy is super scary for granted, but I’m just happy and trust your colleagues who are going to perform that” (subject 11, pre-intervention). Sometimes, patients asked the oncologists for their advice what to do with regards to the decision whether or not to participate in early phase clinical trials, or to other decisions (e.g. whether or not to use certain pain medication, or to get vaccinated against COVID-19). Others referred to different healthcare professionals with whom they have a good (or bad) relationship and who could help them in this decision (e.g. their referring oncologist or general practitioner). In the post-intervention, some patients mentioned that they appreciated the rationale behind a specific trial or that they believed it to be a step in the right direction
Autonomy	In both study phases, autonomy was generally not something that patients explicitly mentioned to consider important, but a value that arose from their expressions and/or actions. Several patients spontaneously indicated that they want to let everything sink in and think about the decision. Sometimes they (also) asked what action they have to take themselves once they have made a decision. A few patients referred to information they actively sought for, or to situations where they took matters in their own hands (e.g. to start directly with hormonal tablets after a surgery instead of waiting as the doctor suggested, or to have actively sought to be referred for early phase clinical trials). Patients sometimes discussed situations that illustrate that they are still independent. Occasionally, patients seemed to have made an (autonomous) decision before the consultation, or they indicated their decision during the consultation in response to the doctor, both to participate (“Yes, I’m definitely in favour [of participation]. Yes, yes, I’ll just go for it”, subject 08, pre-intervention) and not to participate (“Well, hearing this from you, I won’t do it [participate]”, subject 31, post-intervention). No patients mentioned that they gained a sense of control by participating, but sometimes they mentioned that they could decide for themselves if they wanted to stop at any time
Social adherence	When a patient made a remark regarding social adherence, they mostly stated that they find it important to discuss the information and deliberation of the options with their partner/children/other family and friends. Sometimes they mentioned the burden of the early phase clinical trial on the relative, for instance because “[they] are the one who has to drive” (subject 26, post-intervention). One patient mentioned that her husband is also ill, and that she wants to take care of him. Another patient stated that he wants to take care of his wife (by moving to a smaller house) so she can keep living there when he is gone. Relatives that were present during the consultation sometimes gave their opinion about what option they think is best for the patient. However, no patient mentioned to feel pressured by others with regards to his/her decision
Altruism	Patients in the pre-intervention and post-intervention occasionally referred to altruism. If they did so, it was usually by stating that they want to help future other patients, or help develop future treatments by participating in an early phase clinical trial. In some of these cases, they mentioned that they hope for some benefit for themselves, but if they do not, “then let it help science” (subject 22, post-intervention). A patient and relative in the post-intervention justified this belief a bit further by stating he has a relatively unknown tumour type and that “without research, they [healthcare professionals] can never continue [new treatments]” (subject 18, post-intervention)
Corporeality	When asked about their complaints, some patients emphasized the contrast between how ‘good’ they feel and the bad news they recently received (i.e. that there are no standard treatment options available anymore): “I am completely healthy […] apart from having cancer” (subject 28, post-intervention). When patients did feel ill, they sometimes expressed their discontent with the way pain and other tumour-related symptoms affect their daily life. Many patients worried that the cancer will grow and that their condition will deteriorate, especially if ‘nothing’ is done – which seemed to relate to perseverance. Besides, (sometimes the very same) patients expressed that they have had relatively good experiences with previous treatments (e.g. little side effects, appropriate blood levels), with which they seemed to suggest that this will also apply (i.e. that their body will hold up) if they participate in an early phase clinical trial – which seemed to relate to risk tolerance. Some patients express their wish to maintain a healthy lifestyle, for example “And I eat healthy and, since I heard in 2017 [that I have cancer], I haven’t drunk any alcohol” (subject 14, pre-intervention), either because they want to reduce their symptoms or because they hope it will improve their quality/quantity of life. In contrast, other patients described how they care less about maintaining a healthy lifestyle: “I began exercising before last year when it became clear that it [cancer] had returned. Yes well, then I let go of all exercises, I thought, get lost, I’m done with it” (subject 11, pre-intervention). Some patients, however, stated that they are willing to change these habits if it is required for participation in early phase clinical trials
Accepting one’s fate	Patients often stated that they “know that [they’re] going to start the last phase of [their] life” (subject 18, post-intervention). Some patients also mentioned that they accept this fate, while others simply stated that they are aware of the situation, implicitly suggesting some level of acceptance. In contrast, other patients and relatives said that they have a hard time accepting this fate: “can’t accept it for now, because there are still so many things” (relative of subject 03, pre-intervention). Religious and spiritual faith (in general and as a potential means to accept one’s fate) were not mentioned by patients in this study
Humanity	Patients rarely explained that they want to be treated as a human-being (rather than e.g. a patient, number or guinea pig). In the pre-intervention phase, a patient mentioned that others “are all so compassionate and all” (subject 03, pre-intervention) and send lots of messages. Another patient said that his “youngest daughter did not really ask about it [his situation]. And that is okay” (subject 13, pre-intervention). A third patient explicitly mentioned that “[he does] not feel like being turned inside out again” (subject 02, pre-intervention) in order to participate in an early phase clinical trial. Although it is difficult to determine the underlying value in these previous examples, the fact that they brought such matters up seems to indicate that these patients value humanity. Also in the post-intervention, one relative indicated concern for the patient becoming a “guinea pig” (subject 29, post-intervention). Another patient told the oncologist that “it is alright” (subject 29, post-intervention) if he is treated as a number (instead of a name) during teleconferences regarding an early phase clinical trial

**Fig. 1:**
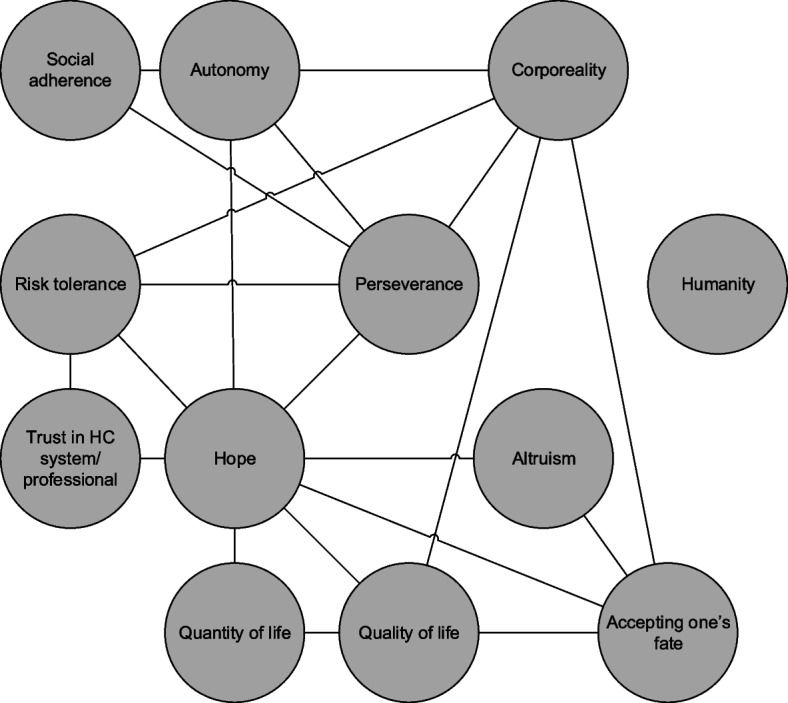
Which patient values were discussed (together) in the pre-intervention phase* * Disclaimer: during the coding of the transcripts/consultations, we encoded the entire phrase (e.g. with the preceding and consecutive sentence, or question/answer) in which a value was mentioned. This figure only provides a visualisation of values that occurred together in these phrases

Several values were referred to in (almost) all pre-intervention discussions: quality of life was mentioned by all patients, and e.g. corporeality, risk tolerance and hope by most. In contrast, other values were mentioned only rarely, such as humanity, altruism, social adherence and trust. Figure 1 illustrates the main links that occurred between values in the pre-intervention phase. For instance, quality of life was mentioned together with accepting one’s fate (“It can of course just be a part of the process […] but I would still like it if I would just feel a little better”, subject 02), corporeality (“I’m fine […] You can stay put, but then you will become a ‘vegetable’, so I still have a reasonable stamina”, subject 03) and quantity of life (“I won’t live for many more years, I think […] I am already living more extensively, but yes, I think that I have to do that”, subject 09). Furthermore, hope and perseverance appeared to be connected, e.g. “Cons, I don’t really see because if you don’t do it, you’re certain that it’s over […] so I can actually only have certain benefits from it [trial participation]” (subject 03). These values were also sometimes referred to together with risk tolerance (as in the example), corporeality, social adherence, autonomy, altruism or trust. The only value that was not mentioned with any of the other values in the pre-intervention phase, was humanity (e.g. one patient mentioned that “[he did] not feel like being turned inside out again”, subject 02).

**Fig. 2:**
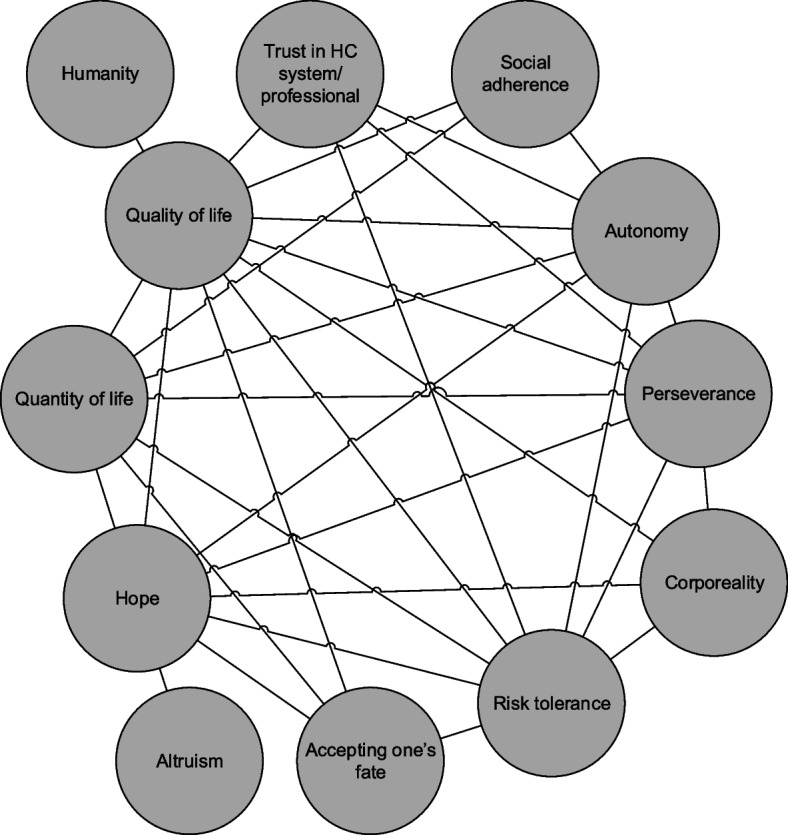
Which patient values were discussed (together) in the post-intervention phase* * Disclaimer: during the coding of the transcripts/consultations, we encoded the entire phrase (e.g. with the preceding and consecutive sentence, or question/answer) in which a value was mentioned. This figure only provides a visualisation of values that occurred together in these phrases

In the post-intervention phase, similarly, quality of life was mentioned by all patients. Other values that were often referred to were risk tolerance, corporeality, autonomy and hope, whereas humanity, altruism and accepting one’s fate were, again, only occasionally mentioned. Figure 2 shows the main links between values in the post-intervention phase. Now, nearly all values were mentioned together with other values, including humanity, which was sometimes mentioned together with quality of life (e.g. “It still has to be humane, that is what I think. And I still enjoy everything” subject 19). Quality of life, hope, risk tolerance, quantity of life, autonomy and perseverance were mentioned along with all other values. There were multiple occasions in which several diverse values were discussed, for example:

*“Of course the biggest question is, what can it bring me, mean to me? Yes, I’m actually already a little happy that I’m hearing from you that I have access to these, at least possible access to these studies. That already relieves a little tension. But well, besides, yes, I hope it helps me […] I have already hoped for four years that science catches up with the disease, and yes, that seems to be less likely and that is hard” (subject 22).*

In this phrase, the patient consecutively referred to the values risk tolerance, trust, hope and accepting one’s fate. Furthermore, some patients very clearly weighed several values against each other, as illustrated in the following quote in which the patient particularly weighed quantity against quality of life:

*“So then you’re talking about an prolongation of life of maybe a few months […], but if that’s it, then I don’t necessarily have to live six months longer. I prefer to have sufficient quality of life. It’s not about the duration of, well that too of course, but […] if I have to choose between the two, I would rather die half a year sooner” (subject ****29****).*

There were a few relations between values that appeared in both the pre-intervention and post-intervention phase: quality of life was sometimes mentioned together with quantity of life or with corporeality; risk tolerance with trust or with hope; hope was also mentioned with perseverance or with altruism; and social adherence with autonomy.

### How patient values were discussed

Based on the consultations, we distinguished four major ways in which patient values were discussed (see Supplementary Table 1 and below).

#### Questions and answers on patient values

Oncologists rarely asked direct questions about patients’ values at the beginning of the pre-intervention conversations. They sometimes indicated that the question “What do I want?” was important, but that it would be left for the time being and discussed later in the consultation. Occasionally, the oncologists explicitly asked the patient “What is important for you to strive for when we discuss treating the illness or not?” (oncologist A) or what they considered to be of importance in general. When patients mentioned values in the beginning of the consultation, they usually did so briefly, in response to a question from the oncologist about their cancer-related symptoms (treatments) and their current physical condition. Patients often mentioned how their cancer (treatment) had affected or was still affecting certain aspects of their life, such as hobbies, work, family and physical exercise. Sometimes patients shortly discussed their values in reaction to more open-ended questions, such as “How can I help you today?” (oncologist D). After mentioning values this early in the consultation, most oncologists continued with asking about the patients’ other (physical) symptoms, medical history or medication use, e.g. “Okay, clear. Do you have any complaints at the moment? How are you doing with eating?” (oncologist C). Later in the consultation, after providing information on early phase clinical trials, some oncologists asked patients for their (first) thoughts, e.g. “Now you have heard all of this, what do you think?” (oncologist I). Few patients then gave responses related to their values, usually by simply indicating what they had hoped for, e.g. “I had hoped that there was an experimental possibility” (subject 13). Oncologists sporadically asked a follow-up question. Besides, oncologists occasionally reacted to patients’ values by stating that it was clear what the patient meant or by saying that “these are really good considerations” (oncologist F).

In the post-intervention phase, similar to pre-intervention phase, oncologists sometimes indicated early in the consultation “What do I want” was an important question that would be addressed later. Furthermore, the discussion of patients’ symptoms and medical history remained an important topic and was, again, sometimes reason for patients to refer to their values. Besides, some oncologists already asked specific questions with regards to what was important to the patients in this phase of their life or “What is your motivation [for participation in an early phase clinical trial]” (oncologist A). In response, patients often described what was important to them in multiple sentences, sometimes explicitly explaining their weighing of values, for instance: “I know it asks a lot of me […] but I think, yes, well, I can get past that […] Only the weeks that I am really sick to death is nasty, but for the rest it’s, well, it’s uncomfortable, but I’m not somebody who says, well, …” (subject 26). In those cases, several oncologists now asked follow-up questions, which enabled a clear dialogue between patient (and relative) and oncologist. For instance, in some cases, the oncologists tried to clarify patients’ weighing of their values by figuring out what they were (not) willing to give up, e.g. “Yes and are there limitations, of which you say, this goes too far for me?” (oncologist A).

#### Continuing the dialogue

Most of the values in the pre-intervention phase were found in short, implicit expressions of patients in response to information from the oncologist about early phase clinical trials. Some patients responded immediately after or even during the information provision, often in the form of questions. For example, patients asked substantive (sometimes multiple or similar) questions about side effects, rather than stating their worries or wishes, e.g. “What are the side effects? […] But is that fatigue really extreme? […] And no other nausea or diarrhoea or…?” (subject 14). When the patient expressed worry about a certain aspect of the treatment, oncologists tried to explain more about the trial. Preferences and worries about the treatment were sometimes discussed at the end of the conversation, when the oncologist asked if the patient (and relatives) had any more questions, or if everything was clear.

In the post-intervention phase, discussions (i.e. in multiple sentences and with (follow-up) questions) about motivation and values often took place after the oncologist shared information about treatment options. Generally, oncologists explicitly mentioned that patients had a choice and that they had to make a well-considered decision based on what was important to them, for instance “And an experimental context comes with a bit of uncertainty, […] So that is a choice that you have to make for yourself […] Is that something that I want” (oncologist A). These remarks were sometimes combined with a concrete question to the patient, such as regarding what the patient thought about the option to participate in an early phase clinical trial. In other cases, patients already spontaneously responded, for instance when an oncologist emphasized the uncertainty of the situation: “You know that you’re starting your last phase of life. I can deal very well with that thus far” (subject 18). In the dialogue that followed, this patient and his relative together described that they wanted to contribute to science, but not if that required too much investment from the patient because that “would be a waste of time”. Similarly, other patients described multiple values in the same passage of the conversation, sometimes clearly providing their weighing towards each other. Some oncologists responded to such considerations by confirming/repeating them, or by asking follow-up questions.

#### Providing two ‘contrasting’ examples

In the pre-intervention phase, the main attempt oncologists made to discuss patient values generally happened after providing information about early phase clinical trials, in the form of an (extensive) story describing two ‘opposite’ examples of patients who accepted or declined early phase clinical trial participation based on different values, e.g.: “One says, guys, I’ve tried my best, […] it’s enough for me. […] And the other person can say, guys, I don’t care, […] if they offer me an inch, I’ll take the entire yard. The optimists, the fighters.” (oncologist D). At times, oncologists thereby mentioned that individual patients can feel differently about it, or that both perspectives can be right. Patients rarely spontaneously indicated how their personal values related to those the oncologist described. After the story, patients were generally asked to reflect about it (and their own thoughts) at home. Sometimes it was suggested to discuss everything with e.g. their close ones or general practitioner, for instance: “You don’t have to decide now, because […] such a decision is a big deal. I can imagine very well that you say, well, I want to think about it for a little while, or think about it together, talk about it with people” (oncologist E).

Some oncologists in the post-intervention phase also used opposite examples similar to those described above. In these occurrences, patients responded by indicating their personal considerations and values.

#### Discussing the OnVaCT

In the post-intervention phase, patient values were sometimes discussed in response to a question by the oncologist about the OnVaCT. Most (but not all) oncologists at least referred to the OnVaCT or the study in general, and often asked patients if they had “thought about it, or about things that are important” (oncologist C) to them personally. A few patients became emotional and had to cry when the OnVaCT was discussed, for instance after saying: “[character from OnVaCT] thought that quality of life, that is important. No longer wanting to keep going at all costs” (subject 21). When the oncologist asked about the OnVaCT, one patient indicated not to have used it, after which the oncologist gave an example of what was described in it and the patient immediately responded with “Yes, that is how I think about it myself” (subject 20). Another patient said that she did not recognize herself in the stories described in the OnVaCT, but when the oncologist asked “But if you had to tell that story for yourself, what is important to you?” (oncologist C), the patient was able to indicate what she missed in the OnVaCT and thereby explain what she valued for herself.

## Discussion

This study aimed to qualitatively explore which (RQ1) and how (RQ2) patient values are discussed before and after the implementation of the OnVaCT intervention in communication about potential participation in early phase clinical cancer trials. Our analysis of consultations shows that similar values arose in the pre-intervention and post-intervention phase, i.e. hope, perseverance, quality or quantity of life, risk tolerance, trust in the healthcare system or professional, autonomy, social adherence, altruism, corporeality, accepting one’s fate, and humanity, with the difference that they were all discussed in context with each other in the post-intervention phase (RQ1). However, the way these values were discussed (i.e. the communication patterns) appeared to differ between the study phases (RQ2). In the pre-intervention phase, patient values were mainly found in short, spontaneous responses of patients to information or explanations provided by the oncologist. In the post-intervention phase, besides similar spontaneous responses, we also found a dialogue between patients and oncologists as well as a concrete weighing of values – often in relation to the OnVaCT. These longer phrases including several values (and their weighing against each other) may explain why almost all values appeared to be connected post-intervention. The implementation of the OnVaCT intervention appears to have changed the doctor-patient consultation both from the perspective of the patient, who talked extensively about values in long paragraphs, as well as from that of the oncologist, who asked concrete (follow-up) questions to explore individual patients’ values and their weighing. These questions may have potentially stimulated patients to tell in-depth about their values.

The results of this study seem to suggest that the intervention may have led to changes in the communication patterns. This would align well with previous studies on decision aids and communication skills trainings. For instance, decision aids (particularly those containing value clarification methods) had shown before to improve e.g. doctor-patient communication and congruence between patients’ informed values and their chosen options [10, 11]. Moreover, communication skills trainings for oncologists can positively impact communication behaviour [39] and SDM [40]. Specifically, assessing patients’ preferences for participation in a phase I clinical trial (e.g. ‘helping other cancer patients by participating in research’, ‘avoiding spending lots of hours in the clinic’) had shown to impact the strength of oncologists’ recommendations [41]. With regards to the OnVaCT intervention, we applied a synergetic approach [42, 43] in which the two different components of the intervention (i.e. the OnVaCT for patients and communication training for oncologists) are intended to enhance each other.

Previous studies have indicated that the discussion of patient values in current standard practice is often limited [19, 27, 28, 44, 45]. Our study is the first to provide a possible nuance to this claim. Already in the pre-intervention phase, we found that several, diverse patient values were (shortly) referred to during the consultation, for instance in response to general-inquiry questions [46–48] from oncologists at the beginning of the consultation (e.g. “how can I help you today?”). Such open-ended questions may encourage patients to “respond in their own terms and permitting the emergence of narratives based in lifeworld experience” [49], thereby exceeding a merely medical-technical discussion. This was also seen in the spontaneous responses from the patient, e.g. during history-taking. In that sense, when patients’ responses reflected their values, they appeared to go beyond the (medical-technical) agenda set by the oncologist [49]. However, oncologists often ignored patients’ explicit mentions or implicit cues [50, 51] on their values by continuing with history-taking questions or information provision, thereby non-explicitly reducing the space for further disclosure [50]. Still, oncologists addressed patient values when they described two opposite examples of what other patients in a similar situation might value. This is an example of ‘detached footing’ [52–55], (e.g. [56]) which may have enabled the oncologist to discuss this difficult topic while keeping a distance, i.e. without ‘labelling’ the patient to have certain values. This way of ‘option-listing’ by the oncologist could help create a moment of choice for their patients [57]. However, rather than asking follow-up questions and starting a dialogue about patients’ values, oncologists in the pre-intervention phase often recommended patients to further reflect at home. By doing so, oncologists explicitly reduced the space for further disclosure [50] of patient values.

Although oncologists seemed to acknowledge the importance of patient values in this complex decision and of following their personal wishes, an actual conversation on patient values was left to the patients and their proxies (e.g. relatives or general practitioner). This approach seems to align best with an ‘informative model’ in which patients are expected to know their personal values, and physicians only provide them with medical-technical information [29] and then offer them the choice to participate in an early phase clinical trial or not (i.e. the ‘logic of choice’ [58]). Still, in SDM, oncologists may be particularly equipped to discuss patient values: who would be better suited to help put the complex information into perspective than those specialized in explaining and conducting such trials, and who saw many patients in their practice? Yet, this required a new approach of the consultation in which oncologists try to understand these patients and discuss what matters to them.

With the OnVaCT intervention, we aimed to facilitate a discussion of patient values between patients and oncologists. The current analysis provides a first indication that the intervention may indeed offer such support. Based on the oncologists’ behaviour, the intervention may have helped them to become more sensitive to patient values, as they asked (follow-up) questions regarding patients’ personal wishes and beliefs. For instance, by asking patients what matters to them. By addressing the identification of patient values and goals of care [59] as a first step of SDM in line with the communication training, oncologists explicitly provided space for further disclosure of patient values [50]. With these probing questions, the oncologists seemed to apply an ‘interpretative model’ [29] in the post-intervention phase, thereby taking on the role of counsellor or advisor to help patients articulate and weigh their values. Moreover, by helping patients to find the right balance in their values, the oncologists may be better able to ‘care’ for these patients (i.e. the ‘logic of care’ [58]) as this approach enables the integration of all aspects of patients and their life with this life-limiting disease. When patients were asked by the oncologists what is important to them (with and without referring to the OnVaCT), most patients were able to explain their values. This even applied when patients did not use or recognize themselves in the OnVaCT narratives: patients who did not use it responded to narratives told by the oncologist, and patients who did not recognize themselves were able to indicate why this was the case and what they personally valued (i.e. what they missed in the OnVaCT). This aligns well with a narrative approach, according to which choices are “part of an effort to live a life that has coherence and meaning” [60]. In other words, decisions will likely have to suit patients’ personal life (stories). By using extended sequences to talk about their values, patients themselves seemed to be telling more of a story on their values too.

When comparing our results to previous studies in the OnVaCT project, there are mainly similarities: nearly all values and (sub-)themes from the systematic review [24] and interview study [26] occurred in clinical practice. Furthermore, similar to the interview study, we found that hope and perseverance were closely related. Regardless, this analysis added two new subthemes (i.e. ‘wanting to avoid inactivity’ under perseverance, and ‘(un)safety of the treatment’ under risk tolerance) to the existing set of values. A more substantial difference was that we did not find any occurrence of the subthemes ‘religious faith and/or being guided by God’ and ‘spiritual faith’ under accepting one’s fate. Besides a potential result of sampling, this could be explained by the fact that religious involvement in the Netherlands has declined over the past decade [61]. Nonetheless, religious or spiritual faith may still be important for at least a substantial minority of patients, which is why we have kept them as subthemes in our codebook.

### Limitations

Although this study offers new insights into the discussion of patient values surrounding early phase clinical trial participation, our findings know some limitations. With the current qualitative content analysis and the study design (i.e. nonrandomized controlled trial), we cannot unquestionably prove that certain observed changes in communication patterns between the study periods are significant, nor that they are a direct result of our intervention. As our findings may have been susceptible to bias [62], they should be interpreted with caution. However, when conducted well, nonrandomized controlled trials can find similar outcomes as randomized controlled trials [63, 64]. The current analysis is, to the best of our knowledge, the first to examine patient values in communication on potential participation in early phase clinical trials, thereby providing initial insights into the clinical practice. In a next part of the OnVaCT project, we will take a closer look into specific SDM steps (including the discussion of patients’ preferences and goals of care) [59, 65], in order to further (quantitatively) explore the relationship of the OnVaCT intervention with SDM and decisional conflict [66].

Furthermore, we did not conduct a discourse analysis [see e.g. 67] nor narrative analysis [see e.g. 68]. Such analyses may be able to build on our findings, for instance by examining what and how language (e.g. in terms of vocabulary, grammar or behaviour) and stories (e.g. types of narratives) are used in this context. The existing discourse and conversation analytic literature that have been referred to above (i.e. [46, 47, 49–55, 57]) may provide inspiration for future analyses. Additionally, topic modelling [69] could be used as a means to verify the suggested links between (phrases of) values.

Lastly, all patients in this study were included in Dutch hospitals and were of Dutch nationality, thereby representing a ‘western world’-perspective. This points to the potentially limited applicability of our findings, since patient values can differ between individuals, countries or cultures. Additionally, we do not know whether maximum variability was achieved, for example in terms of patients’ religious and spiritual beliefs, as we did not include data on this. We still included patients and oncologists from several hospitals across the Netherlands, and aimed for sufficient spread in terms of patients’ decision, age and tumour type to substantiate the diversity of our sample, although only a small proportion of all available consultations were qualitatively analysed. Besides, for some oncologists, there were only limited available recordings in the pre-intervention and/or post-intervention phase. Although we only included oncologists with at least one available recording in both phases, this may have caused some bias in our results based on oncologists’ personal approach of these consultations. Additionally, only the main and/or clearest relations between values were indicated. Notwithstanding the insights these visualisations provide, for instance into which other values patients may be thinking about when they indicate a certain value, some relations may have been missed.

### Implications for practice

Notwithstanding the limitations described above, we believe that the OnVaCT intervention may be of practical relevance for healthcare providers and patients facing this difficult decision together. For instance, the communication training could be used in the education of oncological specializations, such as for oncologists-in-training or research nurses, to help them improve the discussion of patient values as part of SDM for potential participation in early phase clinical trials. Besides, Dutch referring hospitals as well as specialized early phase clinical trial units can (continue to) share the OnVaCT with their patients to help them prepare to clarify their values during the upcoming SDM process. In order to implement the OnVaCT in other countries, sometimes cultural adaptation (especially of the values and narratives) may be required in addition to language translation. Meanwhile, it is important to note that it remains unclear which (or both) of the two intervention parts was responsible for the observed differences between the pre-intervention and post-intervention phase, i.e. what the effective ingredients were. Yet, with the complex SDM process in mind, we are confident that an intervention aimed at patients and oncologists was the only appropriate approach to support both parties in their shared process. For the sake of transparency/open science as well as to enable others to implement the OnVaCT intervention in clinical practice, an elaborate description of the intervention (i.e. a link to the OnVaCT and the documents used in the communication training) has been made available online [35].

## Conclusions

This study has indicated the potential of the OnVaCT intervention (consisting of the OnVaCT for patients and communication training for oncologists) to support the integration of patient values into SDM consultations. In particular, this qualitative analysis has pointed to the different patterns surrounding patient values in communication on potential participation in early phase clinical trials. Besides, the current study provides nuance to previous claims regarding the limited discussion of patient values in oncology practice. All identified patient values were referred to already pre-intervention, with the difference that these values were all discussed in context with each other post-intervention. Furthermore, in the pre-intervention phase, most values were only found in short expressions of patients, e.g. in response to information from the oncologist, while the post-intervention phase again showed different communication patterns, with many oncologists referring to the OnVaCT and asking follow-up questions, and patients using phrases in which multiple values occurred. This analysis thus seems to provide qualitative evidence for the claim that the OnVaCT intervention can support the integration of patient values in consultations for early phase clinical trial participation.

### Supplementary Information

**Additional file1:** **Supplementary table 1.** Final codebook (i.e. additions and changes to the initial codebook are italicized).

## Data Availability

The data analysed during the current study (i.e. transcripts in Dutch) are not publicly available due to privacy and ethical considerations, but are available from the corresponding author on reasonable request.

## Abbreviations

IPDAS: International Patient Decision Aids Standards
MRC: Medical Research Council
OnVaCT: Online Value Clarification Tool
SDM: Shared Decision-Making

## References

[1] Elwyn G, O'Connor A, Stacey D, Volk R, Edwards A, Coulter A, Thomson R, Barratt A, Barry M, Bernstein S (2006). Developing a quality criteria framework for patient decision aids: online international Delphi consensus process. BMJ.

[2] O'Connor AM, Llewellyn-Thomas HA, Flood AB (2004). Modifying Unwarranted Variations In Health Care: Shared Decision Making Using Patient Decision Aids: A review of the evidence base for shared decision making. Health Affairs.

[3] Ebenau A, van Gurp J, Hasselaar J (2017). Life values of elderly people suffering from incurable cancer: A literature review. Patient Educ Couns.

[4] Schwartz SH (2007). Basic human values: Theory, measurement, and applications. Rev Fr Sociol.

[5] Rescher N (1969). Introduction to value theory.

[6] Légaré F, O'Connor AC, Graham I, Saucier D, Côté L, Cauchon M, Paré L (2006). Supporting patients facing difficult health care decisions: use of the Ottawa Decision Support Framework. Can Fam Physician.

[7] O'Connor AM, Tugwell P, Wells GA, Elmslie T, Jolly E, Hollingworth G, McPherson R, Bunn H, Graham I, Drake E (1998). A decision aid for women considering hormone therapy after menopause: decision support framework and evaluation. Patient Educ Couns.

[8] Hoefel L, O’Connor AM, Lewis KB, Boland L, Sikora L, Hu J, Stacey D (2020). 20th anniversary update of the Ottawa decision support framework Part 1: a systematic review of the decisional needs of people making health or social decisions. Med Decis Making.

[9] Stacey D, Legare F, Boland L, Lewis KB, Loiselle M-C, Hoefel L, Garvelink M, O’Connor A (2020). 20th anniversary Ottawa decision support framework: part 3 overview of systematic reviews and updated framework. Med Decis Making.

[10] Hoefel L, Lewis KB, O’Connor A, Stacey D (2020). 20th anniversary update of the Ottawa decision support framework: part 2 subanalysis of a systematic review of patient decision aids. Med Decis Making.

[11] Stacey D, Légaré F, Col NF, Bennett CL, Barry MJ, Eden KB, Holmes‐Rovner M, Llewellyn‐Thomas H, Lyddiatt A, Thomson R (2014). Decision aids for people facing health treatment or screening decisions.

[12] Fagerlin A, Pignone M, Abhyankar P, Col N, Feldman-Stewart D, Gavaruzzi T, Kryworuchko J, Levin CA, Pieterse AH, Reyna V (2013). Clarifying values: an updated review. BMC Med Inform Decis Mak.

[13] Elwyn G, Laitner S, Coulter A, Walker E, Watson P, Thomson R (2010). Implementing shared decision making in the NHS. BMJ.

[14] Weston WW (2001). Informed and shared decision-making: the crux of patient-centred care. CMAJ.

[15] Stiggelbout AM, Van der Weijden T, De Wit MPT, Frosch D, Légaré F, Montori VM, Trevena L, Elwyn G. Shared decision making: really putting patients at the centre of healthcare. Bmj. 2012;344:e256. 10.1136/bmj.e256 22286508

[16] Elwyn G, Frosch D, Thomson R, Joseph-Williams N, Lloyd A, Kinnersley P, Cording E, Tomson D, Dodd C, Rollnick S (2012). Shared decision making: a model for clinical practice. J Gen Intern Med.

[17] Stiggelbout AM, Pieterse AH, De Haes J (2015). Shared decision making: concepts, evidence, and practice. Patient Educ Couns.

[18] Epstein RM, Street RL (2011). Shared mind: communication, decision making, and autonomy in serious illness. The Annals of Family Medicine.

[19] Kunneman M, Marijnen CAM, Baas-Thijssen MCM, van der Linden YM, Rozema T, Muller K, Geijsen ED, Stiggelbout AM, Pieterse AH (2015). Considering patient values and treatment preferences enhances patient involvement in rectal cancer treatment decision making. Radiother Oncol.

[20] Baker A. Crossing the quality chasm: a new health system for the 21st century, vol. 323. British Medical Journal Publishing Group; 2001. 10.17226/10027.

[21] Wolfe A (2001). Institute of Medicine report: crossing the quality chasm: a new health care system for the 21st century. Policy Polit Nurs Pract.

[22] Beach MC, Sugarman J (2019). Realizing shared decision-making in practice. JAMA.

[23] Hoffmann TC, Montori VM, Del Mar C (2014). The connection between evidence-based medicine and shared decision making. JAMA.

[24] van Lent LGG, Jabbarian LJ, van Gurp J, Hasselaar J, Lolkema MP, van Weert JCM, van der Rijt CCD, de Jonge MJA. Identifying patient values impacting the decision whether to participate in early phase clinical cancer trials: A systematic review. Cancer Treat Rev. 2021;98:102217. 10.1016/j.ctrv.2021.102217 33965892

[25] Pentz RD, Flamm AL, Sugarman J, Cohen MZ, Ayers GD, Herbst RS, Abbruzzese JL (2002). Study of the media's potential influence on prospective research participants' understanding of and motivations for participation in a high-profile phase I trial. J Clin Oncol.

[26] van Gurp JLP, van Lent LGG, Stoel NK, van der Rijt CCD, de Jonge MJA, Pulleman SM, van Weert JCM, Hasselaar J. Core values of patients with advanced cancer considering participation in an early-phase clinical cancer trial: a qualitative study. Support Care Cancer. 2022;30:7605–13. 10.1007/s00520-022-07200-5 PMC9385761 35676342

[27] Thorne S, Hislop TG, Kim-Sing C, Oglov V, Oliffe JL, Stajduhar KI (2014). Changing communication needs and preferences across the cancer care trajectory: insights from the patient perspective. Support Care Cancer.

[28] Trice ED, Prigerson HG (2009). Communication in end-stage cancer: review of the literature and future research. J Health Commun.

[29] Emanuel EJ, Emanuel LL (1992). Four models of the physician-patient relationship. JAMA.

[30] Pichler T, Rohrmoser A, Letsch A, Westphalen CB, Keilholz U, Heinemann V, Lamping M, Jost PJ, Riedmann K, Herschbach P (2021). Information, communication, and cancer patients’ trust in the physician: what challenges do we have to face in an era of precision cancer medicine?. Support Care Cancer.

[31] Craig P, Dieppe P, Macintyre S, Michie S, Nazareth I, Petticrew M (2008). Developing and evaluating complex interventions: the new Medical Research Council guidance. BMJ.

[32] Shahsavari H, Matourypour P, Ghiyasvandian S, Nejad MRG (2020). Medical Research Council framework for development and evaluation of complex interventions: a comprehensive guidance. Journal of Education and Health Promotion.

[33] van Gurp JLP, van Lent LGG, Stoel NK, van der Rijt CCD, van Weert JCM, Hasselaar J (2023). Accentuating patient values in shared decision-making: a mixed methods development of an Online Value Clarification Tool and communication training in the context of early phase clinical cancer trials. Patient Educ Couns.

[34] van Lent LGG, Stoel NK, van Weert JCM, van Gurp J, de Jonge MJA, Lolkema MP, Gort EH, Pulleman SM, Oomen-de Hoop E, Hasselaar J (2019). Realizing better doctor-patient dialogue about choices in palliative care and early phase clinical trial participation: towards an online value clarification tool (OnVaCT). BMC Palliat Care.

[35] Online Value Clarification Tool (OnVaCT) with communication training [Dutch title: "Online Value Clarification Tool (OnVaCT) met communicatietraining"] [https://www.healthcommunication.nl/blog/online-value-clarification-tool-onvact-met-communicatietraining/]

[36] O’Brien BC, Harris IB, Beckman TJ, Reed DA, Cook DA (2014). Standards for Reporting Qualitative Research: A Synthesis of Recommendations. Acad Med.

[37] Newell DJ (1992). Intention-to-treat analysis: implications for quantitative and qualitative research. Int J Epidemiol.

[38] Hsieh H-F, Shannon SE (2005). Three Approaches to Qualitative Content Analysis. Qual Health Res.

[39] Barth J, Lannen P (2011). Efficacy of communication skills training courses in oncology: a systematic review and meta-analysis. Ann Oncol.

[40] Henselmans I, van Laarhoven HWM, van Maarschalkerweerd P, de Haes HCJM, Dijkgraaf MGW, Sommeijer DW, Ottevanger PB, Fiebrich HB, Dohmen S, Creemers GJ (2020). Effect of a skills training for oncologists and a patient communication aid on shared decision making about palliative systemic treatment: a randomized clinical trial. Oncologist.

[41] Hianik RS, Owonikoko T, Switchenko J, Dixon MD, Shaib WL, Pentz RD (2021). Evaluating the impact of the Patient Preference Assessment Tool on clinicians' recommendations for phase I oncology clinical trials. Psychooncology.

[42] Brandes K, Linn AJ, Butow PN, Weert J (2015). The characteristics and effectiveness of Question Prompt List interventions in oncology: a systematic review of the literature. Psychooncology.

[43] Linn AJ, van Weert JCM, Smit EG, Perry K, van Dijk L (2013). 1+ 1= 3? The systematic development of a theoretical and evidence-based tailored multimedia intervention to improve medication adherence. Patient Educ Couns.

[44] Thomas TH, Jackson VA, Carlson H, Rinaldi S, Sousa A, Hansen A, Kamdar M, Jacobsen J, Park ER, Pirl WF (2019). Communication differences between oncologists and palliative care clinicians: a qualitative analysis of early, integrated palliative care in patients with advanced cancer. J Palliat Med.

[45] Cripe LD, Vater LB, Lilly JA, Larimer A, Hoffmann ML, Frankel RM (2021). Goals of care communication and higher-value care for patients with advanced-stage cancer: A systematic review of the evidence.

[46] Heritage J, Robinson JD (2006). The structure of patients' presenting concerns: physicians' opening questions. Health Commun.

[47] Robinson JD, Heritage J (2006). Physicians’ opening questions and patients’ satisfaction. Patient Educ Couns.

[48] Robinson JD, Heritage J (2014). Intervening with conversation analysis: The case of medicine. Res Lang Soc Interact.

[49] Boyd E, Heritage J (2006). uestioning during comprehensive history-taking. In: Communication in medical care: Interaction between primary care physicians and patients.

[50] Del Piccolo L, De Haes H, Heaven C, Jansen J, Verheul W, Bensing J, Bergvik S, Deveugele M, Eide H, Fletcher I (2011). Development of the Verona coding definitions of emotional sequences to code health providers’ responses (VR-CoDES-P) to patient cues and concerns. Patient Educ Couns.

[51] Zimmermann C, Del Piccolo L, Bensing J, Bergvik S, De Haes H, Eide H, Fletcher I, Goss C, Heaven C, Humphris G (2011). Coding patient emotional cues and concerns in medical consultations: the Verona coding definitions of emotional sequences (VR-CoDES). Patient Educ Couns.

[52] Teas Gill V, Maynard DW (1995). On “labeling” in actual interaction: Delivering and receiving diagnoses of developmental disabilities. Soc Probl.

[53] Monzoni CM, Reuber M, O'Reilly M (2016). Psychogenic non-epileptic seizures: how doctors use medical labels when they communicate and explain the diagnosis. The Palgrave handbook of adult mental health: discourse and conversation studies.

[54] Stortenbeker I, Stommel W, van Dulmen S, Lucassen P, Das E, Olde Hartman T (2020). Linguistic and interactional aspects that characterize consultations about medically unexplained symptoms: A systematic review. J Psychosom Res.

[55] Stortenbeker I, Stommel W (2022). olde Hartman T, van Dulmen S, Das E: How General Practitioners Raise Psychosocial Concerns as a Potential Cause of Medically Unexplained Symptoms: A Conversation Analysis. Health Commun.

[56] Monzoni C, Reuber M. Linguistic and interactional restrictions in an outpatient clinic. Producing and Managing Restricted Activities. 2015. p. 239–70.

[57] Toerien M, Reuber M, Shaw R, Duncan R (2018). Generating the perception of choice: the remarkable malleability of option-listing. Sociol Health Illn.

[58] Mol A (2008). The logic of care: Health and the problem of patient choice.

[59] Pel-Littel RE, Buurman BM, van de Pol MH, Yilmaz NG, Tulner LR, Minkman MM (2019). op Reimer WJMS, Elwyn G, van Weert JCM: Measuring triadic decision making in older patients with multiple chronic conditions: Observer OPTIONMCC. Patient Educ Couns.

[60] Nicholas B, Gillett G (1997). Doctors' stories, patients' stories: a narrative approach to teaching medical ethics. J Med Ethics.

[61] Religion in the Netherlands (Dutch title: Religie in Nederland) [https://www.cbs.nl/nl-nl/longread/statistische-trends/2020/religie-in-nederland]

[62] Aggarwal R, Ranganathan P (2019). Study designs: Part 4–interventional studies. Perspect Clin Res.

[63] Ioannidis JPA, Haidich A-B, Pappa M, Pantazis N, Kokori SI, Tektonidou MG, Contopoulos-Ioannidis DG, Lau J (2001). Comparison of evidence of treatment effects in randomized and nonrandomized studies. JAMA.

[64] Mathes T, Rombey T, Kuss O, Pieper D (2021). No inexplicable disagreements between real-world data–based nonrandomized controlled studies and randomized controlled trials were found. J Clin Epidemiol.

[65] Elwyn G, Edwards A, Wensing M, Grol R (2005). Shared decision making measurement using the OPTION instrument.

[66] O'Connor AM (1995). Validation of a decisional conflict scale. Med Decis Making.

[67] Brown G, Yule G (1983). Discourse analysis.

[68] Bury M (2001). Illness narratives: fact or fiction?. Sociol Health Illn.

[69] Vayansky I, Kumar SAP (2020). A review of topic modeling methods. Inf Syst.

