# Supplementary material for: Patient values in patient-provider communication about participation in early phase clinical cancer trials: a qualitative analysis before and after implementation of an online value clarification tool intervention
Source: BMC Med Inform Decis Mak. 2024 Feb 2;24:32. doi: 10.1186/s12911-024-02434-1 (PMC10835819; doi:10.1186/s12911-024-02434-1)
Supplement: Supplementary file 1 — Additional file1: Supplementary table 1. Final codebook (i.e. additions and changes to the initial codebook are italicized). [file 12911_2024_2434_MOESM1_ESM.docx]

Supplementary table 1

*Final codebook (i.e. additions and changes to the initial codebook are italicized)*

| **Patient values and subthemes** | **Definition** (based on review and interview study) |
| --- | --- |
| **Hope**   - Hope for personal benefit - Having an optimistic attitude | The desire, belief or feeling that participating in an early phase clinical cancer trial will lead to personal benefit (such as tumour shrinkage or prolongation of life). Hope and optimism are often used as synonyms. |
| **Perseverance**   - Wanting to ‘fight/battle’ cancer - Wanting to have tried everything - *Wanting to avoid inactivity** | The desire to keep fighting/battling (or to keep living), despite having little treatment options or a bad prognosis. |
| **Quality or quantity of life**   - Quality of life - Quantity of life | The preferences someone has regarding the balance and potential trade-off between living as long as possible and maintaining quality of life *(e.g. absence of complaints and/or negative side effects)*. |
| **Risk tolerance**   - Wanting to take a gamble/risk (by participating in a trial) - Regular/close follow-up - *(Un)safety of the treatment** | The willingness or ability someone has to accept risks or take gambles. |
| **Trust in the healthcare system or healthcare professional**   - Trust in medicine - Communicating trust in the healthcare professional | The belief that a healthcare professional or institution (including his/her judgement and endorsement) is good, sincere, and/or honest and that he/she would not willingly trick or harm someone. |
| **Autonomy**   - Gaining a sense of control by participating - Wanting to make a decision for oneself - Wanting to be/stay/act independent | The desire or ability to act and make decisions without being controlled by and/or dependent on others in the context of a life-limiting disease. |
| **Social adherence**   - Feeling pressure from others (family/friends) - Wanting to follow the wishes of others (family/friends) | The desire or willingness to behave according to the expectations, values or attitudes of others (especially family) regarding participation in an early phase clinical cancer trial. |
| **Altruism**   - Wanting to help future patients - Wanting to help research/medicine | The desire or willingness to accept or decline participation in an early phase clinical cancer trial motivated by the care for others (including wanting to help research/medicine), even though it does not necessarily lead to personal benefit. |
| ***Corporeality* (previously: body preservation)****   - Maintaining a healthy lifestyle - Relaxing/experiencing pleasure (unhealthy behaviour does not matter anymore) - (Dis)trusting one’s body | The relation with the body changes when being diagnosed with cancer. What people find most important; creating the optimal environment to succeed treatment by living healthy or seeing the body as a medium for enjoyment has a determining role in the decision-making process |
| **Accepting one’s fate**   - *(by means of)* Religious faith and/or being guided by God*** - *(by means of)* Spiritual faith*** | The willingness or ability someone has to accept the things, especially bad things such as nearing death and/or worsening complaints, that will happen to him/her. In this sense, religion and spirituality may serve as means to help accept such a fate. |
| **Humanity**   - Not wanting to be a ‘guinea pig’ - Wanting to be treated as human, rather than a patient - Wanting others to show interest in them (and their illness/family/etc) | The desire to be, feel, or be treated as a person rather than a god, an animal, a machine, or a patient. |
| **Attempt of the doctor to start discussion/deliberation of value(s)**   - *Questions and answers on patient values** - *Continuing the dialogue** - *Providing two ‘opposite’ examples** - *Discussing the (results from the) OnVaCT** | An effort the oncologist makes to start or continue a discussion or deliberation with the patient regarding his/her values (e.g. with questions or examples). N.B. This is not considered a ‘patient value’ but was included in the codebook to better enable the unravelling of how these values were discussed. |

** These subthemes were added during/based on the data analysis*

*** We changed ‘body preservation’ into ‘corporeality’ as we believed patients’ descriptions to go beyond simply preserving their body (generally in favour of early phase clinical trial participation), as they also addressed actually using their body while they can (e.g. for going to work, enjoying a beer or hobbies).*

**** These subthemes were not mentioned in the recordings, but were kept as subthemes as they may be of relevance to future studies (e.g. that also include data on patients’ religious or spiritual beliefs).*
